# Impact of COVID-19 lockdown on physical activity and sedentary behaviour in Dutch cardiovascular disease patients

**DOI:** 10.1007/s12471-021-01550-1

**Published:** 2021-02-25

**Authors:** B. M. A. van Bakel, E. A. Bakker, F. de Vries, D. H. J. Thijssen, T. M. H. Eijsvogels

**Affiliations:** 1 Department of Physiology, Radboud Institute for Health Sciences, Radboud University Medical Centre, Nijmegen, The Netherlands; 2 Research Institute for Sports and Exercise Sciences, Liverpool John Moores University, Liverpool, UK

**Keywords:** COVID-19, SARS-CoV‑2, Cardiovascular disease, Physical activity, Sedentary behaviour, Prevention

## Abstract

**Background:**

Coronavirus disease 2019 (COVID-19) lockdown restrictions may impact lifestyle and therefore also physical (in)activity patterns in patients with cardiovascular disease (CVD). This study aimed to evaluate the effect of lockdown on physical activity and sedentary behaviour.

**Methods:**

A total of 1565 Dutch CVD patients participated in this prospective cohort study, in which we compared physical activity and sedentary behaviour before and during the COVID-19 lockdown period. Baseline measures were assessed in 2018 and data on follow-up measures were collected between 17 and 24 April 2020 (5 weeks after the introduction of COVID-19 lockdown restrictions). Validated questionnaires were used to assess physical activity and sedentary behaviour.

**Results:**

Moderate-to-vigorous physical activities increased from 1.6 (0.9, 2.8) to 2.0 (1.0, 3.5) h/day [median (interquartile range)] (*p* < 0.001) during the COVID-19 lockdown, mainly due to an increase in time spent walking and doing odd jobs. In contrast, time spent exercising significantly declined [1.0 (0.0, 2.3) to 0.0 (0.0, 0.6) h/week], whereas sedentary time increased from 7.8 (6.1, 10.4) to 8.9 (6.8, 11.4) h/day (*p* < 0.001). The absolute increase in physical activity was 13 (−36, 81) min/day, whereas sedentary behaviour increased by 55 (−72, 186) min/day.

**Conclusion:**

Despite a small increase in physical activities, the larger increase in sedentary time induced a net reduction in habitual physical activity levels in Dutch CVD patients during the first-wave COVID-19 lockdown. Since a more inactive lifestyle is strongly associated with disease progression and mortality, we encourage CVD patients and their caregivers to explore novel solutions to increase physical activity levels and reduce sedentary time during (and beyond) the COVID-19 pandemic.

## What’s new?

- A net reduction in habitual physical activity levels was found in chronic cardiovascular disease (CVD) patients during the first-wave coronavirus disease 2019 (COVID-19) lockdown in the Netherlands; the increase in sedentary time was greater than the increase in physical activity.
- A more sedentary lifestyle may induce greater detrimental health effects in patients with CVD than in the general population due to their lower habitual activity levels.
- Telerehabilitation initiatives may be considered to increase physical activity patterns among chronic CVD patients during, but also beyond, the COVID-19 pandemic.

## Background

Lockdown procedures have been instituted worldwide to limit the spread of severe acute respiratory syndrome coronavirus‑2 (SARS-CoV-2), but social distancing restrictions may impact lifestyle behaviour, including physical activity and sedentary behaviour in the home environment, commuting and leisure-time activities [1, 2]. Large between-country differences exist in the extent of measures to prevent the spread of coronavirus disease 2019 (COVID-19), whilst strong differences may also occur concerning how these measures affect an individual’s physical activity pattern. Since cardiovascular disease (CVD) patients are at increased risk for severe COVID-19 complications [3], they may be highly compliant with social distancing restrictions and, consequently, vulnerable as regards the impact of these restrictions on physical activity patterns [4]. To critically explore the impact of the lockdown measures in the Netherlands, our study aimed to compare physical activity and sedentary behaviour in a large group of Dutch CVD patients before and during the COVID-19 lockdown.

## Methods

CVD patients participating in an ongoing multicentre study (*n* = 2178) [5] were invited via e‑mail to complete an online questionnaire. The study was performed in four Dutch hospitals (i.e. Radboud University Medical Centre, Jeroen Bosch Hospital, Rijnstate Hospital and Isala Clinic) in collaboration with the Dutch Heart Foundation. Patients were included if they were diagnosed with CVD and/or were eligible for cardiac rehabilitation based on the guidelines of the American Heart Association [6] and the European Society of Cardiology [7]. Data on baseline measures were collected in April-October 2018 and on follow-up measures between 17 and 24 April 2020 (i.e. 36 days after the introduction of lockdown restrictions). Dutch lockdown measures included closure of public facilities, bars, restaurants, schools and sports clubs and the recommendation to stay at and work from home. When outside, social distancing had to be adhered to, with the restriction not to meet more than one other person at any one time. In our questionnaire, we assessed lockdown adherence by the question to what extent patients adhered to the COVID-19 lockdown restrictions, using a 10-point Likert scale, with 0 representing no adherence and 10 representing complete adherence. Validated questionnaires were used to assess physical activity [8] and sedentary behaviour [9] concerning the 7 days preceding study participation, whereas general characteristics and health status were collected at baseline and follow-up. Incomplete questionnaires were excluded from further analyses. Daily time spent performing moderate-to-vigorous intensity physical activity (MVPA) was determined in four different domains: work, transportation, household and leisure time. Leisure-time activities consisted of walking, cycling, doing odd jobs (e.g. doing repairs, washing the car, painting a room), gardening and performing exercise. We assessed the time spent sedentary in nine distinct everyday life situations that can be stratified into work, transportation and leisure time. Medical ethical approval was obtained (#2020-6414) at the Radboud University Medical Centre, and all patients provided informed consent.

Data were reported as number (%) for categorical variables and as median (interquartile range) for non-normally distributed continuous variables. All parameters were checked for normality using the Shapiro-Wilk test. Differences in physical activity and sedentary behaviour between the lockdown period and the control period were tested using a Mann-Whitney U test for non-normally distributed continuous variables. We calculated the percentage of patients with decreased MVPA, decreased exercise time and increased sitting time with a cumulative percentage of patients with deterioration in any of these physical activity domains. Stratified and multivariate linear regression analyses were performed to examine whether changes in physical activity and sedentary behaviour were impacted by age, sex, CVD subtypes, lockdown adherence and baseline levels of physical activity, exercise training and sedentary behaviour. All statistical tests were two-sided and significance was set at *p* < 0.05. Analyses were performed with IBM SPSS Statistics 25 (IBM Corp., Armonk, NY, USA).

## Results

**Fig. 1:**
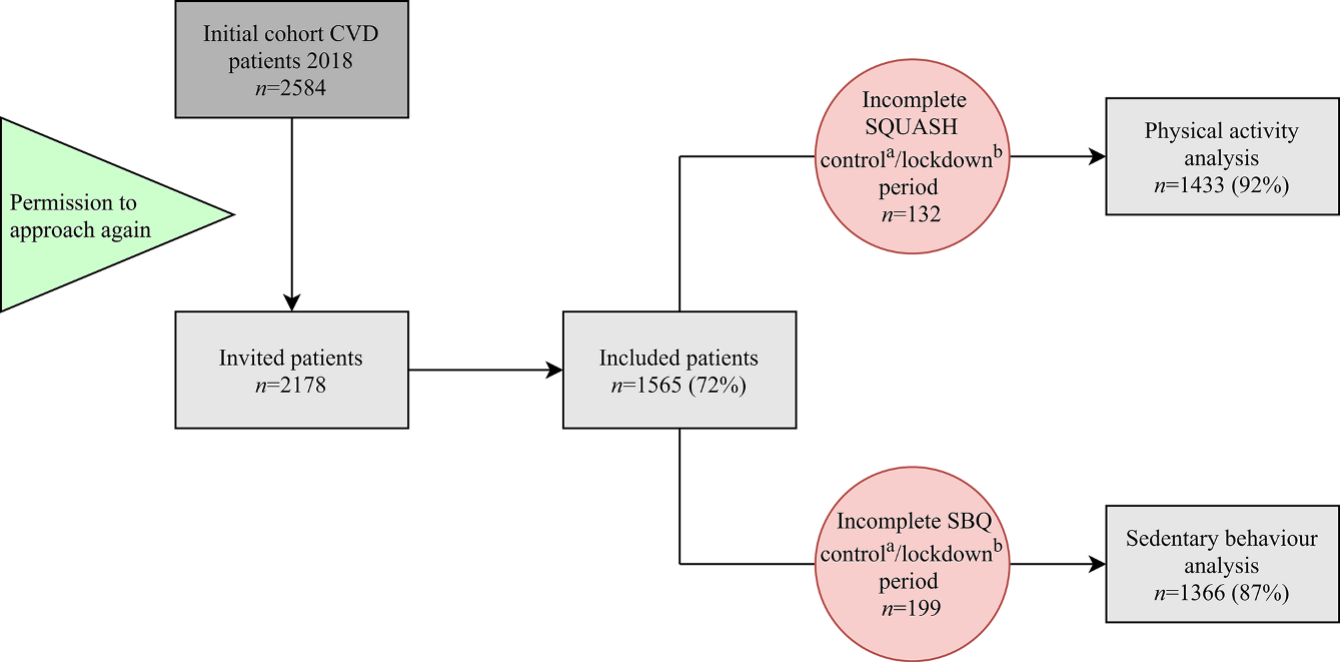
Study flowchart. In total, 2178 patients were approached, of whom 1565 (72%) participated in the present study: 1433 (92%) completed the baseline and follow-up questionnaire regarding physical activity and 1366 (87%) that for sedentary behaviour. *CVD* cardiovascular disease, *SBQ* sedentary behaviour questionnaire, *SQUASH* short questionnaire to assess health-enhancing physical activity. ^a^Control period: April-October 2018. ^b^Lockdown period: 17–24 April 2020 (36 days after introduction of COVID-19 lockdown measures in the Netherlands)

In total, 1565 patients participated in the present study (response rate 72%), of whom 1433 (92%) completed the baseline and follow-up questionnaire regarding physical activity and 1366 (87%) that for sedentary behaviour (Fig. 1). All continuous variables were non-Gaussian distributed [i.e. MVPA, sitting time, age, body mass index (BMI) and lockdown adherence]. Participants were 65 (58, 70) years, more often male (73%), overweight or obese (68%) and mostly retired (51%). Most patients were diagnosed with myocardial infarction (48.2%) or angina pectoris (18.1%) (Tab. 1). The characteristics of participants did not differ from those of non-participants (data not shown). On the 10-point scale, adherence to the lockdown restrictions was reported as 9 [8, 10]. Overall, 42% of CVD patients decreased MVPA, 45% reduced exercise time, and 62% increased sedentary time during the COVID-19 lockdown. A combination of these outcomes revealed that 84.6% of participating CVD patients either decreased physical activity, reduced exercise time, and/or increased sedentary behaviour.

**Table 1:** Baseline characteristics

Patient characteristics		*n* = 1565	Missing values
Age (years)		65 (58, 70)	0 (0%)
Sex (male)		1144 (73.1%)	0 (0%)
BMI (kg/m^2^)		26.5 (24.4, 29.1)	136 (9%)
Lockdown adherence		9 (8, 10)	4 (0.3%)
Employment status (retired)		804 (51.4%)	1 (0.1%)
CVD subtype^a^			0 (0%)
	Myocardial infarction	754 (48.2%)	
	Angina pectoris	284 (18.1%)	
	Heart valve disease	139 (8.9%)	
	Heart failure	119 (7.6%)	
	Other^b^	269 (17.2%)	

Data were reported as *n* (%) or median (interquartile range)

*BMI* body mass index; *CVD* cardiovascular disease

^a^CVD subtype was based on main diagnosis at the time of the study period (2020)

^b^Other was defined as heart rhythm disorders, congenital heart disease, stroke and peripheral artery disease

### Physical activity

**Fig. 2:**
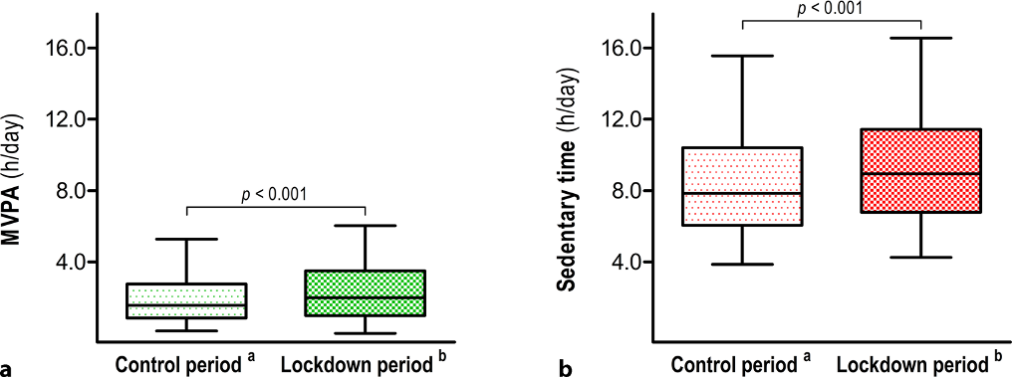
Boxplots of moderate-to-vigorous physical activity (*MVPA*, **a**) and sedentary time (**b**) during the control period and COVID-19 lockdown period. Despite a small increase in time spent performing MVPA [+0.2 (−0.6, 1.4) h/day] during the COVID-19 lockdown period compared to the control period, a larger increase in sedentary time [+0.9 (−1.2, 3.1) h/day] was observed, leading to a net reduction in habitual physical activity levels. Boxplots represent median, interquartile range and 5th and 95th percentiles. ^a^Control period: April-October 2018. ^b^Lockdown period: 17–24 April 2020 (36 days after introduction of COVID-19 lockdown measures in the Netherlands)

MVPA increased from 1.6 (0.9, 2.8) to 2.0 (1.0, 3.5) h/day (*p* < 0.001) during the COVID-19 lockdown (Fig. 2), which was largely due to changes in leisure time [∆: 0.3 (−0.4, 1.3) h/day], with almost no changes in activities at work [∆: 0.0 (0.0, 0.0)], during transportation [∆: 0.0 (0.0, 0.0)] or in the household [∆: 0.0 (0.0, 0.1)]. Increases in leisure-time MVPA were due to walking [0.3 (0.0, 0.6) to 0.4 (0.0, 1.0)] and doing odd jobs [0.0 (0.0, 0.3) to 0.0 (0.0, 1.0)], but not cycling [∆: 0.0 (−0.2, 0.1)] and gardening [∆: 0.0 (0.0, 0.1)]. The absolute increase in physical activity was 13 (−36, 81) min/day. Time spent exercising significantly declined [1.0 (0.0, 2.3) to 0.0 (0.0, 0.6) h/week]. Stratified analysis showed no significant increase in MVPA in women [1.4 (0.8, 2.4) to 1.5 (0.7, 2.6) h/day (*p* = 0.39)], patients with heart valve disease [1.6 (0.8, 2.7) to 1.8 (0.9, 3.2) h/day (*p* = 0.11)], and patients with higher levels of MVPA (> 1.6 h/day) at baseline [2.8 (2.1, 3.9) to 2.9 (1.6, 4.4) h/day (*p* = 0.14)] (Tab. 2). Further, changes in MVPA were not impacted by age, BMI, lockdown adherence, exercise training or sedentary time at baseline.

**Table 2:** Comparison of moderate-to-vigorous physical activity and sedentary time between the control period and COVID-19 lockdown period across different patient- and behavioural characteristics

			Moderate-vigorous physical activity (h/day) *n* = 1433				Sedentary time (h/day) *n* = 1366			
			*n* (%)	Control period^a^	Lockdown period^b^	*p*-value	*n* (%)	Control period^a^	Lockdown period^b^	*p*-value
*Patient characteristics*										
	Sex									
		Females	381 (27)	1.4 (0.8, 2.4)	1.5 (0.7, 2.6)	0.39	359 (26)	7.6 (5.9, 10.0)	8.8 (6.8, 11.4)	<0.001
		Males	1052 (73)	1.7 (0.9, 2.9)	2.1 (1.1, 3.7)	<0.001	1007 (74)	7.9 (6.1, 10.5)	9.0 (6.7, 11.4)	<0.001
	*Age (years)*									
		≤65	756 (53)	1.5 (0.9, 2.5)	2.0 (1.0, 3.5)	<0.001	724 (53)	8.5 (6.4, 11.2)	9.3 (7.1, 12.0)	<0.001
		>65	677 (47)	1.8 (0.9, 2.9)	2.0 (1.0, 3.5)	<0.001	642 (47)	7.4 (5.6, 9.2)	8.5 (6.4, 10.8)	<0.001
	*BMI (kg/m*^*2*^*)*									
		Normal (<25)	456 (32)	1.7 (0.9, 2.7)	2.1 (1.0, 3.7)	<0.001	435 (32)	7.3 (5.9, 9.8)	8.5 (6.6, 10.8)	<0.001
		Overweight (≥25)	973 (68)	1.6 (0.9, 2.8)	1.9 (0.9, 3.4)	<0.001	927 (68)	8.1 (6.2, 10.6)	9.1 (6.9, 11.6)	<0.001
	*CVD subtype*^c^									
		Myocardial infarction	753 (53)	1.6 (0.9, 2.8)	2.0 (1.0, 3.4)	<0.001	719 (53)	7.8 (5.9, 10.6)	9.0 (6.8, 11.5)	<0.001
		Angina pectoris	278 (19)	1.8 (1.1, 3.0)	2.1 (1.0, 3.9)	0.006	267 (20)	7.9 (6.2, 10.0)	8.9 (6.6, 11.1)	<0.001
		Heart valve disease	137 (10)	1.6 (0.8, 2.7)	1.8 (0.9, 3.2)	0.11	130 (10)	7.5 (5.6, 9.9)	8.5 (6.8, 10.8)	<0.001
		Heart failure	119 (8)	1.3 (0.8, 2.1)	1.4 (0.6, 3.2)	0.010	114 (8)	8.4 (6.3, 11.6)	9.2 (6.3, 12.3)	0.07
		Other^d^	146 (10)	1.5 (0.9, 2.7)	2.1 (0.9, 3.7)	0.001	136 (10)	8.2 (6.7, 10.2)	8.6 (7.0, 11.8)	0.002
Behavioural characteristics										
	*Lockdown adherence*									
		≤8/10	400 (28)	1.8 (1.0, 2.9)	2.0 (1.0, 3.5)	<0.001	376 (28)	8.2 (6.1, 10.6)	9.0 (6.8, 11.2)	<0.001
		>8/10	1031 (72)	1.6 (0.9, 2.7)	2.0 (1.0, 3.5)	<0.001	989 (72)	7.7 (6.0, 10.4)	8.9 (6.8, 11.5)	<0.001
	*Exercise training (h/week)*									
		≤1	830 (58)	1.4 (0.7, 2.5)	1.9 (0.9, 3.4)	<0.001	795 (58)	7.8 (6.0, 10.4)	8.8 (6.5, 11.3)	<0.001
		>1	603 (42)	1.9 (1.2, 3.1)	2.0 (1.1, 3.7)	0.001	571 (42)	8.0 (6.1, 10.5)	9.2 (7.1, 11.5)	<0.001
	*MVPA (h/day)*									
		≤1.6	717 (50)	0.9 (0.5, 1.2)	1.3 (0.6, 2.4)	<0.001	681 (50)	8.2 (6.4, 11.0)	9.3 (7.2, 12.0)	<0.001
		>1.6	716 (50)	2.8 (2.1, 3.9)	2.9 (1.6, 4.4)	0.14	685 (50)	7.5 (5.7, 9.8)	8.6 (6.4, 10.9)	<0.001
	*Sedentary time (h/day)*									
		≤7.8	703 (49)	1.7 (0.9, 3.1)	2.1 (1.1, 3.7)	<0.001	676 (49)	6.0 (4.8, 7.0)	7.7 (5.7, 9.5)	<0.001
		>7.8	730 (51)	1.5 (0.8, 2.5)	1.8 (0.9, 3.4)	<0.001	690 (51)	10.4 (8.9, 12.4)	10.5 (8.3, 13.1)	0.13

Data were reported as *n* (%) or median (interquartile range)

*BMI* body mass index, *CVD* cardiovascular disease, *MVPA* moderate-vigorous physical activity

^a^Control period: April-October 2018

^b^Lockdown period: 17–24 April 2020 (36 days after introduction of COVID-19 lockdown measures in the Netherlands)

^c^CVD subtype was based on main diagnosis at the time of the study period (2020)

^d^Other was defined as heart rhythm disorders, congenital heart disease, stroke and peripheral artery disease

### Sedentary behaviour

Sedentary time increased from 7.8 (6.1, 10.4) to 8.9 (6.8, 11.4) h/day (*p* < 0.001) during lockdown (Fig. 2). Sitting during leisure time increased by 1.2 (−0.4, 3.1) h/day, mainly due to television viewing [2.0 (1.1, 3.0) to 2.4 (2.0, 3.3)] and listening to music [0.1 (0.0, 0.8) to 0.3 (0.0, 1.0)], whereas sitting during transportation decreased by −0.3 (−0.7, 0.0) h/day. Sitting time at work did not change. The absolute increase in sedentary time was 55 (−72, 186) min/day. Stratified analysis showed no significant increase in sedentary time in patients with heart failure [8.4 (6.3, 11.6) to 9.2 (6.3, 12.3) h/day (*p* = 0.07)] and patients with high levels of sitting time (> 7.8 h/day) at baseline [10.4 (8.9, 12.4) to 10.5 (8.3, 13.1) h/day (*p* = 0.13)] (Tab. 2). Changes in sedentary time were not impacted by age, BMI, lockdown adherence, exercise training or MVPA at baseline, but females (*p* = 0.046) demonstrated greater increases in sitting time.

## Discussion

In our prospective study, we found in a large group of Dutch chronic CVD patients that daily physical activity slightly increased [13 (−36, 81) min/day], but that the absolute increase in sedentary behaviour was much greater [55 (−72, 186) min/day], highlighting a net decrease in habitual physical activity patterns during the initial weeks of the COVID-19 lockdown in the Netherlands. Given the deleterious health effects of a sedentary lifestyle in the long term, novel solutions to increase physical activity levels and reduce sedentary time during (and beyond) the COVID-19 pandemic are warranted.

To our knowledge, this is the first prospective study to assess changes in physical activity and sedentary behaviour in chronic CVD patients before versus during the COVID-19 lockdown using longitudinal measurements. The increase in physical activity and simultaneous rise in sedentary time are in line with findings in the general population [10], possibly explained by an increased population-level interest in both physical activity and television viewing during the COVID-19 lockdown [11]. The increase in habitual physical activity was explained by more time spent walking and doing odd jobs, while time spent exercising significantly decreased. The decrease in exercise time was in line with findings of a recent study in which 45.1% of French patients with chronic coronary syndromes (*n* = 195) reported a more than 25% reduction in physical activity [12]. Our joint findings indicate that the lockdown limited exercise possibilities to a large extent.

The net increase in time spent sedentary is alarming, since the combination of low levels of physical activity and high levels of sedentary behaviour are strongly related to adverse outcomes, including disease progression [13] and all-cause mortality [14, 15]. Moreover, a recent systematic review and meta-analysis found that any type of MVPA has beneficial effects on immunisation after vaccination, especially in older adults [16]. Regular MVPA also improves human immunity to infectious diseases, potentially limiting the impact of COVID-19 or other future pandemics [16]. This highlights the need to facilitate engagement in physical activity and reduction of sedentary behaviour in patients with chronic CVD, especially during COVID-19 lockdown restrictions.

The present study assessed only the acute effects of the COVID-19 lockdown on (in)activity patterns, raising the question as to how such behaviour would change following a prolonged lockdown and/or lifting the restrictions. A recent big data study among 455,404 participants from the general population across the world used smartphone step count measurements to determine trends in activity patterns [17]. As expected, a reduction in step count was observed after the lockdown, but no complete recovery was observed after the COVID-19 restrictions were lifted, highlighting the possibility that habitual physical activity demonstrates long-term disturbances following initial lockdown measures. The ongoing second wave and associated second lockdown (as of 14 October 2020) in the Netherlands further increase the possibility that activity patterns of CVD patients will be affected, also beyond the pandemic.

A potential solution to increase physical activity levels and reduce time spent sitting could be to offer lifestyle interventions to chronic CVD patients. Such programmes are typically coordinated by cardiac rehabilitation centres. Among the available interventions, telerehabilitation has several benefits over centre-based rehabilitation. For example, lockdown restrictions are not violated as patients can exercise at home and do not need to travel to the hospital [18]. Furthermore, telerehabilitation can be offered relatively simply and affordably to a large, heterogeneous group using video or telephone consultations, text messaging and social media [18]. It has, therefore, been suggested that the availability of cardiac telerehabilitation should be upscaled during the current COVID-19 pandemic and that these (online) services also be made (freely) available to chronic CVD patients [18, 19].

Telerehabilitation is not restricted to online material only, but can be combined with telephone consultations or written educational material if needed. Such alternative approaches may be valuable to specific subgroups of CVD patients. Indeed, although changes in physical activity and sedentary behaviour were similar in younger and older adults in our study, it has been reported that older adults less often use online tools to perform exercise compared to younger individuals, potentially due to a lower level of digital literacy [10]. Additionally, other studies have found that older adults are more adherent to social distancing [20, 21], indicating that adherence to lockdown restrictions may be more pronounced in individuals at higher risk for COVID-19 complications. These findings emphasise that a tailored approach should be chosen to improve physical activity levels and reduce sedentary time across the heterogeneous population of CVD patients.

Certain limitations apply to our study, including the fact that the assessment of physical activity and sedentary behaviour was based on self-reported data rather than objective measurements, as well as the heterogeneity of CVD diagnosis. However, we performed longitudinal measurements using existing data regarding the same period 2 years ago as our baseline measurement, in contrast to retrospective studies in which reporting bias is likely. Subjective assessment is prone to lead to over- or underestimation of physical activity and sedentary behaviour, respectively [22]. Nevertheless, these questionnaires have good reproducibility, meaning that our repeated measurement design allowed us to validly examine changes in physical activity patterns within patients across time. Another important advantage of subjective assessment compared to objective measurements is that the former facilitates domain-specific information regarding physical activity and sedentary behaviour. This allowed us to specifically identify domains associated with higher sedentary behaviour (e.g. television viewing), and contributed to better understanding of the increase in lower-to-moderate physical activity patterns in Dutch CVD patients during the COVID-19 lockdown measures. Adherence to lockdown measures is a comprehensive concept. Although we did not distinguish between specific components of lockdown adherence, such as social distancing, working from home or avoiding large groups of people, in general our participants reported that they respected the overall lockdown restrictions. Furthermore, weather conditions could have been different between the control and the lockdown period. However, based on Dutch Meteorological Institute (KNMI) data, no major differences were found regarding environmental temperature (16 °C vs 11 °C), rainfall (47 vs 11 mm/month) and average hours of sunshine (229 vs 287 h/month) during the control versus the lockdown period. Hence, we expect that the effect of weather conditions on physical activity levels is only minor, if not negligible.

One strength of our study is that we performed stratified analyses for different subgroups. Changes in physical activity and sedentary behaviour were not impacted by age, BMI, lockdown adherence or exercise training at baseline. However, females, patients with heart valve disease and patients with higher baseline levels of physical activity did not increase their physical activity, while patients with heart failure and patients with high levels of sedentary behaviour at baseline showed no significant increase in sitting time during the COVID-19 lockdown. Identifying subgroups that show a deterioration in physical activity patterns during lockdown enables a targeted approach to encourage CVD patients to adhere to an active lifestyle and thereby prevent health risks associated with physical inactivity.

## Conclusion

The large majority of chronic CVD patients showed a deterioration in physical activity patterns as a result of COVID-19 lockdown restrictions. Since these patients are at increased risk for recurrent cardiovascular events and mortality, interventions are needed to increase physical activity levels and reduce sedentary time in their daily life. Development and implementation of telerehabilitation have gained momentum, providing scalable services to a large, heterogeneous group of patients. These platforms may also be used to offer digital approaches to chronic lifestyle management of CVD patients, which is important given the observations in our study pertaining to the acute and long-term detrimental effects of COVID-19 lockdown restrictions on (in)activity patterns of chronic CVD patients.
